# Supplementary material for: Selection of primary health care quality indicators in Europe: A Delphi study protocol
Source: PLoS One. 2024 Oct 24;19(10):e0309395. doi: 10.1371/journal.pone.0309395 (PMC11500873; doi:10.1371/journal.pone.0309395)
Supplement: S2 File — (PDF) [file pone.0309395.s003.pdf]

Primary care quality indicators - a European Delphi study

Consent to participate in the Delphi study

* Indica uma pergunta obrigatória

What is the purpose of the study?

Primary health care (PHC) plays a central role in the provision of health care, contributing to health promotion and disease prevention. It differs from other care settings by providing continuous care in an integrated manner that is focused on the whole person, according to each patient's unique needs, culture, values, and preferences in the context of their family and community. Being the patient's first contact with the health system, PHC practices coordinate care within the health system, across other specialty care and hospitals.

Regular monitoring plays a critical role to ensure and improve the quality of PHC, where health care indicators are essential to raise awareness, transparency, accountability, and to guide and assess health reforms. Despite efforts to develop and adopt performance indicators in PHC, the abundance of indicators complicates their utilization. Moreover, different stakeholders look for different information and even if the same aspects of care are assessed, the indicator can be valued differently. Therefore, developing a set of indicators requires considering the views of potential users as well as the specific context of a particular health system. The aim of this study is to select a set of quality indicators to assess and compare the quality of PHC based on their importance and validity as perceived by general practitioners and public health physicians, nurses, and other healthcare professionals, as well as researchers and other stakeholders in healthcare quality assessment.

What will I be asked to do if I take part?

The Delphi Process will seek to obtain consensus on the opinions of 'experts', through structured questionnaires or 'rounds' and controlled feedback.

In each round, you will have to rate the importance and validity of 20 to 50 quality indicators, which have been previously abstracted from the scientific literature (https://doi.org/10.1371/journal.pone.0220888) and reviewed by our team of experts. We anticipate on average three-minutes of your time per indicator to complete each round.

For your convenience, the rounds will be administered using the eDelphi software – a Delphi method online tool, which will allow you to work through the indicators at your own pace, stopping and resuming according to your own time.

After the initial round, you will have the opportunity to reconsider your ratings in a second questionnaire that will include both individual and the group's ratings. The impact of any changes in your ratings will be viewed in real time. In practice, you will be able to modify your ratings ideally until a group consensus is reached. In order to allow a timely conclusion of the study, we would respectfully request a response time of 2 weeks for the completion of each round.

There are no right or wrong answers as the study is seeking your opinion. We hope you will find the process interesting and the results will be made available to you at the end of the study.

Who is organizing and funding the research?

This study is part of the 1st.IndiQare project “Quality indicators in primary health care: validation and implementation of quality indicators as an assessment and comparison tool”, funded by FEDER - Fundo Europeu de Desenvolvimento Regional funds through the COMPETE 2020 - Operacional Programme for Competitiveness and Internationalisation (POCI), and by Portuguese funds through FCT - Fundação para a Ciência e a Tecnologia. The Delphi study will be conducted by Mariana Lobo, a post-doc research fellow at the Faculty of Medicine, University of Porto (FMUP) (Portugal), and supervised by Alberto Freitas, an Assistance Professor at FMUP.

Confidentiality

Your personal information (name, email) will be collected strictly to communicate with you throughout the Delphi process. Your country of residence/work, field of expertise (GP, public health, nursing, other) and demographic information will be used to analyze the results of the Delphi process. Survey responses will be collated anonymously using a fictitious identification number without access to the re-identification key. All responses received in the study will be strictly confidential, and your identity will not be divulged. Direct quotes to free-text answers may be used as part of the study report or later Delphi iterations, but these will not be traceable back to you.

Data protection

Questionnaire responses will be anonymized. Data will be stored for the duration of the research project only and then deleted. You have the right to access submitted information during the period of the study.

Questions, Comments or Concerns

If you have any questions or concerns please do not hesitate to contact us by e-mail.
delphigeral2122@gmail.com

1. I consent to participate in this Delphi study, to develop a validated list of primary health care (PHC) quality indicators in European countries. *

   Marcar apenas uma oval.

   ◯ Yes

   ◯ No

2. Name *

   ____

3. E-mail *

   ____

4. Country of residence/work *

Marcar apenas uma oval.

- Albania
- Andorra
- Armenia
- Austria
- Azerbaijan
- Belarus
- Belgium
- Bosnia and Herzegovina
- Bulgaria
- Croatia
- Cyprus
- Czech Republic
- Denmark
- Estonia
- Finland
- France
- Georgia
- Germany
- Greece
- Hungary
- Iceland
- Ireland
- Israel
- Italy
- Kazakhstan
- Kosovo
- Kyrgyzstan
- Latvia
- Lithuania
- Luxembourg
- Malta
- Monaco
- Montenegro
- Netherlands

- North Macedonia
- Norway
- Poland
- Portugal
- Republic of Moldova
- Romania
- Russian Federation
- San Marino
- Serbia
- Slovakia
- Slovenia
- Spain
- Sweden
- Switzerland
- Tajikistan
- Turkey
- Turkmenistan
- Ukraine
- United Kingdom
- Uzbekistan
- Other non-European country

5. Professional area *

Marcar apenas uma oval.

- General practitioner/Family doctor
- Public health physician
- Nurse
- Other healthcare professional
- Researcher
- Outra: ____________________

6. Preferred clinical contexts of quality indicators to be assess in this Delphi study (Please choose according to your expertise; if you are comfortable with PHC indicators, please choose "all")

Marcar tudo o que for aplicável.

- [ ] A - General and unspecified
- [ ] D - Digestive tract
- [ ] F - Eyes
- [ ] H - Ears
- [ ] K - Cardiovascular
- [ ] L - Musculoskeletal
- [ ] P - Psychological
- [ ] R - Respiratory
- [ ] T - Endocrine/Metabolic and Nutritional
- [ ] U - Urological
- [ ] W - Pregnancy and family planning
- [ ] X - Female Genital / Y - Male Genital

Este conteúdo não foi criado nem aprovado pela Google.

Google Formulários
