# Supplementary material for: Selection of primary health care quality indicators in Europe: A Delphi study protocol
Source: PLoS One. 2024 Oct 24;19(10):e0309395. doi: 10.1371/journal.pone.0309395 (PMC11500873; doi:10.1371/journal.pone.0309395)
Supplement: S3 File — (PDF) [file pone.0309395.s004.pdf]

Primary care quality indicators - a European Delphi study

Reasons for declining participation

1. Please select the reason(s) that made you decline your participation in the Delphi study

Check all that apply.

- [ ] Lack of time
- [ ] Longer than anticipated time response
- [ ] Defficient study description
- [ ] Issues in study design
- [ ] Lack of expertise
- [ ] On-line program not intuitive/easy to use
- [ ] Issues using the on-line program
- [ ] Other: ____________________

This content is neither created nor endorsed by Google.

Google Forms
