# Supplementary material for: Selection of primary health care quality indicators in Europe: A Delphi study protocol
Source: PLoS One. 2024 Oct 24;19(10):e0309395. doi: 10.1371/journal.pone.0309395 (PMC11500873; doi:10.1371/journal.pone.0309395)

Primary care quality indicators - a European Delphi study

Information sheet

Thank you for having accepted to participate in this Delphi study. This information sheet aims to provide some brief guidance on what you are expected to do in this Delphi study. However, if you have any questions throughout the process, do not hesitate to contact us at delphigeral2122@gmail.com, marianalobo@med.up.pt, (please use "[Delphi]" followed by the number of your panel on the subject of the email to help us provide prompt feedback).

We encourage you to watch the short demonstration video (here) explaining the process of participation in this Delphi study.

What is the purpose of this study?

Primary health care (PHC) plays a central role in the provision of health care, contributing to health promotion and disease prevention. It differs from other care settings by providing continuous care in an integrated manner that is focused on the whole person, according to each patient's unique needs, culture, values, and preferences in the context of their family and community. Being the patient's first contact with the health system, PHC practices coordinate care within the health system, across other specialty care and hospitals.

Regular monitoring plays a critical role to ensure and improve the quality of PHC, where health care indicators are essential to raise awareness, transparency, accountability, and to guide and assess health reforms. Despite efforts to develop and adopt performance indicators in PHC, the abundance of indicators complicates their utilization. Moreover, different stakeholders look for different information and even if the same aspects of care are assessed, the indicator can be valued differently. Therefore, developing a set of indicators requires considering the views of potential users as well as the specific context of a particular health system.

The aim of this study is to select a set of quality indicators (QIs) to assess and compare the quality of PHC based on their importance and validity as perceived by potential users and considering the context of health systems in European countries.

What is Quality of Care?

In 1999 the National Academy of Medicine, former Institute of Medicine, defined quality of care as "the degree to which health services increase the likelihood of desired results and are consistent with current professional knowledge," and categorized the domains of health quality in 2001 into six pillars: safe, effective, efficient, timely, patient-centered, and equitable [1,2].

What are health indicators?

Health indicators are an important instrument for any healthcare system to improve performance and the quality of the health care provided to patients/users. These consist of measurable elements of the structures, processes, and outcomes of care and are usually operationalized as a ratio between a numerator and a denominator specifying the population at risk.[3]

Preparation of candidate indicators – Pre-Delphi

The PHC QIs under evaluation in this Delphi were essentially abstracted from the scientific literature, those identified in an umbrella review of systematic reviews covering PHC QIs and in the primary studies of the reviews included in this umbrella.[4] In total, 1726 indicators were identified covering different types of indicators (e.g. outcome, process, and structure), capturing different types (e.g. acute, chronic, preventive) and purposes (e.g. screening, diagnosis, treatment, follow up) of care, as well as, different domains of quality of care, and covering several clinical contexts (i.e. WHO ICPC-2 chapters categorization[5]).

However, several of these QIs lacked a proper definition, such as numerator and denominator, and were sometimes rather concepts of measurement. Therefore, in preparation for this Delphi process, a group of researchers from our team completed the characterization of indicators by proposing formulas to all indicators, which were then reviewed by 6 independent medical doctors.

The final list of candidate QIs has been ordered by context and then distributed across **several** Delphi panels (**you will only take part in one of these panels**). Each panel will comprise 10 experts/participants who will have to assess between 20 to 50 indicators. In most cases, all the indicators assigned to one panel correspond to only one clinical context (e.g. Respiratory system) to make the evaluation more meaningful by comparison of similar QIs. Though, generally, not all indicators of that context will be included in one single panel. There are a few exceptions in this distribution of QIs across panels, in which QIs of different contexts were combined in one panel because the number of retrieved QIs from the literature per some contexts was small.

The Delphi process

A Delphi process seeks to obtain consensus on the opinions of 'experts', through a series of structured questionnaires. As part of the process, the responses from each round are fed back in summarized form to the participants, who are then given the opportunity to respond again to the emerging data. The Delphi is, therefore, an iterative multi-stage process designed to combine opinion into group consensus.

[1] Institute of Medicine (US) Committee on Quality of Health Care in America. Improving the 21st-century Health Care System. Crossing the Quality Chasm: A New Health System for the 21st Century. Washington, DC: National Academies Press; 2001.

[2] Wolfe A. Institute of Medicine Report: crossing the quality chasm: a new health care system for the 21st century. Policy Polit Nurs Pract. 2001; 2(3):233-235. doi:10.1177/152715440100200312

[3] Ramalho A, Castro P, Gonçalves-Pinho M, et al. Primary health care quality indicators: an umbrella review. PLoS One. 2019;14(8):e0220888. doi:10.1371/journal.pone.0220888

[4] Jan Mainz, Defining and classifying clinical indicators for quality improvement, International Journal for Quality in Health Care, Volume 15, Issue 6, December 2003, Pages 523–530, https://doi.org/10.1093/intqhc/mzg081

[5] WONCA–World Organization of Family Doctors. An introduction to the international classification of primary care. Version 2" at http://www.ph3c.org/PH3C/docs/27/000098/0000054.pdf Accessed on 05 Jul 2019

How to answer?

In each round, you will have to rate the importance and validity of approximately 25 QIs, which have been previously abstracted from the scientific literature and reviewed by a team of experts. We anticipate on average three minutes of your time per indicator to complete each round.

For your convenience, the rounds will be administered using the eDelphi software – a Delphi method online tool, which will allow you to work through the indicators at your own pace, stopping and resuming according to your own time. Your ratings may be changed at any time while the panel is open. After the initial round, you will have the opportunity to reconsider your ratings in a second questionnaire (2nd round) that will include both your and the group's ratings.

In order to allow a timely conclusion of the study, we would respectfully request a **response time of 2 weeks for the completion of each round**.

There are no right or wrong answers as the study is seeking your opinion. The **participation is anonymous**, we cannot link your answers to you and you can drop out at any time.

We hope you will find the process interesting and the results will be made available to you at the end of the study.

Rating criteria and scale

In each round, you will be asked to rate QIs according to two criteria.

Essentially, you will have to rate how much you agree with two statements, on a scale from **1 – highly disagree** to **9 – highly agree**.

IMPORTANCE

Definition: The indicator should capture important performance aspects of primary health care. It should address areas of concern of policymakers, where there is a clear gap and potential for improvement through identifiable events associated with the healthcare system. It should also permit useful comparisons within and across countries.

Rating: Rate the indicator according to how much you agree/disagree with the statement:

The indicator is an important health quality indicator.

VALIDITY

Definition: The indicator is valid if it is clinically logical and supported by consensus (i.e. face validity); represents a valid measure supported by evidence demonstrating a correlation with the quality of care (i.e. content validity); is reproducible by and comparable across different countries, organizations, and providers, as well as using different data sources over time (i.e. reliable/consistent); detects important changes in the quality of care discriminating well different levels healthcare quality (i.e. sensitivity and specificity).

Rating: Rate the indicator according to how much you agree/disagree with the statement:

The indicator is a valid health quality indicator.

Consensus definition

Consensus will be determined based on the distribution of participants' ratings and the level of disagreement among the participants.

- Indicators with 70% or more ratings in the **1-3** range and less than 15% of ratings in the 7-9 range, will be classified as "**Not important**" or "**Not valid**", depending on the criteria being evaluated.
- Indicators with 85% or more ratings in the **4-6** range, will be classified as "**Uncertain**".
- Indicators with 70% or more ratings in the **7-9** range and less than 15% of ratings in the 1-3 range, will be classified as "**Important**" or "**Valid**", depending on the criteria being evaluated.

Delphi rounds

The Delphi process will end when panel consensus has been achieved or 3 rounds have been completed.

The first round will be carried as a traditional Delphi round to ensure anonymity and prevent biased ratings.

The following rounds will be carried in real-time to support an interactive and efficient process that ensures effective discussions between participants. In practice, when you provide your ratings and comments, you will be immediately confronted with the aggregated results of all other experts' answers who have participated thus far.

All rounds will allow for asynchronous answering so that one expert/participant can take part several times and change his or her answers until the end of a given time frame (minimum two weeks) is reached.

An indicator will be removed from subsequent rounds depending on the number of criteria that reached consensus and the type of consensus reached according to the table 1.

Table 1. Decision matrix to determine which indicators transition to subsequent rounds.

			IMPORTANCE			
			Consensus			No consensus
			Not important	Uncertain	Important	
VALIDITY	Consensus	Not valid	remove	remove	remove	remove
		Uncertain	remove	remove	keep	keep
		Valid	remove	keep	remove	keep
	No consensus		remove	keep	keep	keep

Questionnaire structure and QI presentation

To access the questionnaire from the frontal page of the panel, click on “START ANSWERING” or “CHANGE ANSWERS” or click on the name of the round. Background documents are provided on the left side of this page, under the “DOCUMENTS” tab. Bulletins will be made available throughout the Delphi process presenting the summary of results.

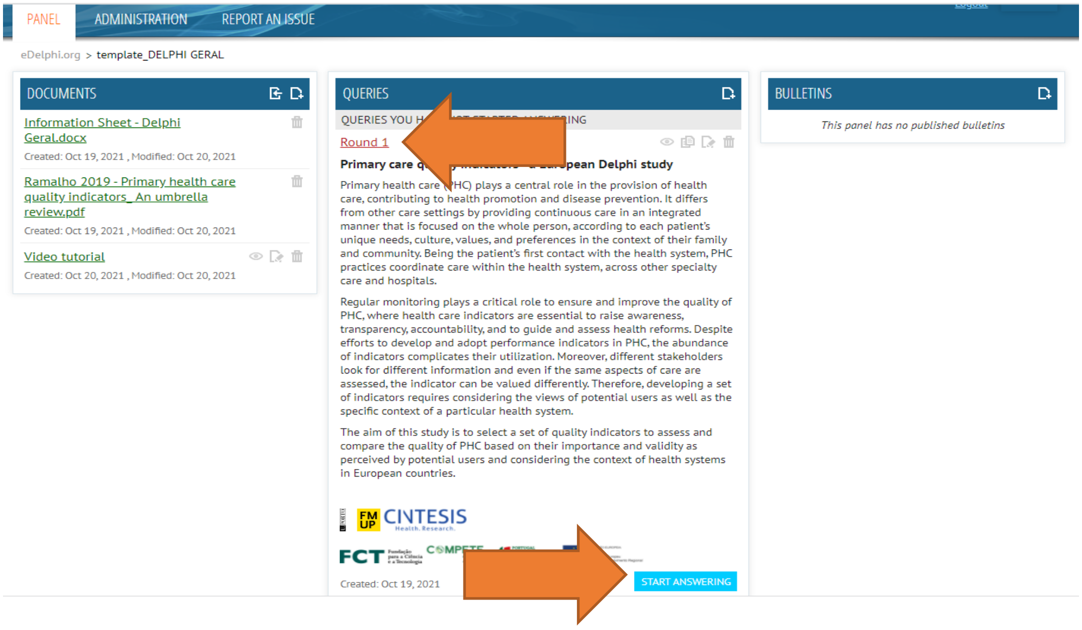

The questionnaire itself is composed of several pages. The initial page provides brief information on how to answer the questionnaire, that is how to rate QIs.

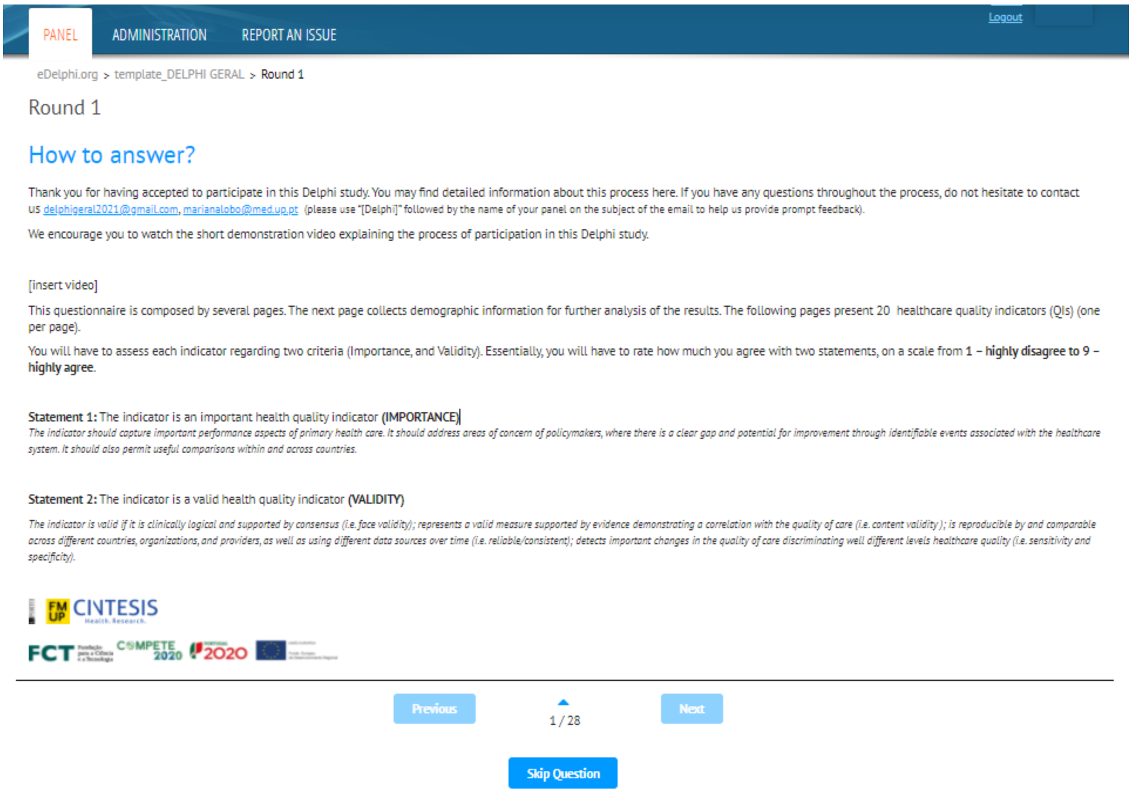

The second page was designed to collect demographic information of the panel to use for further analysis of ratings. It is **important** to select the options that apply to you.

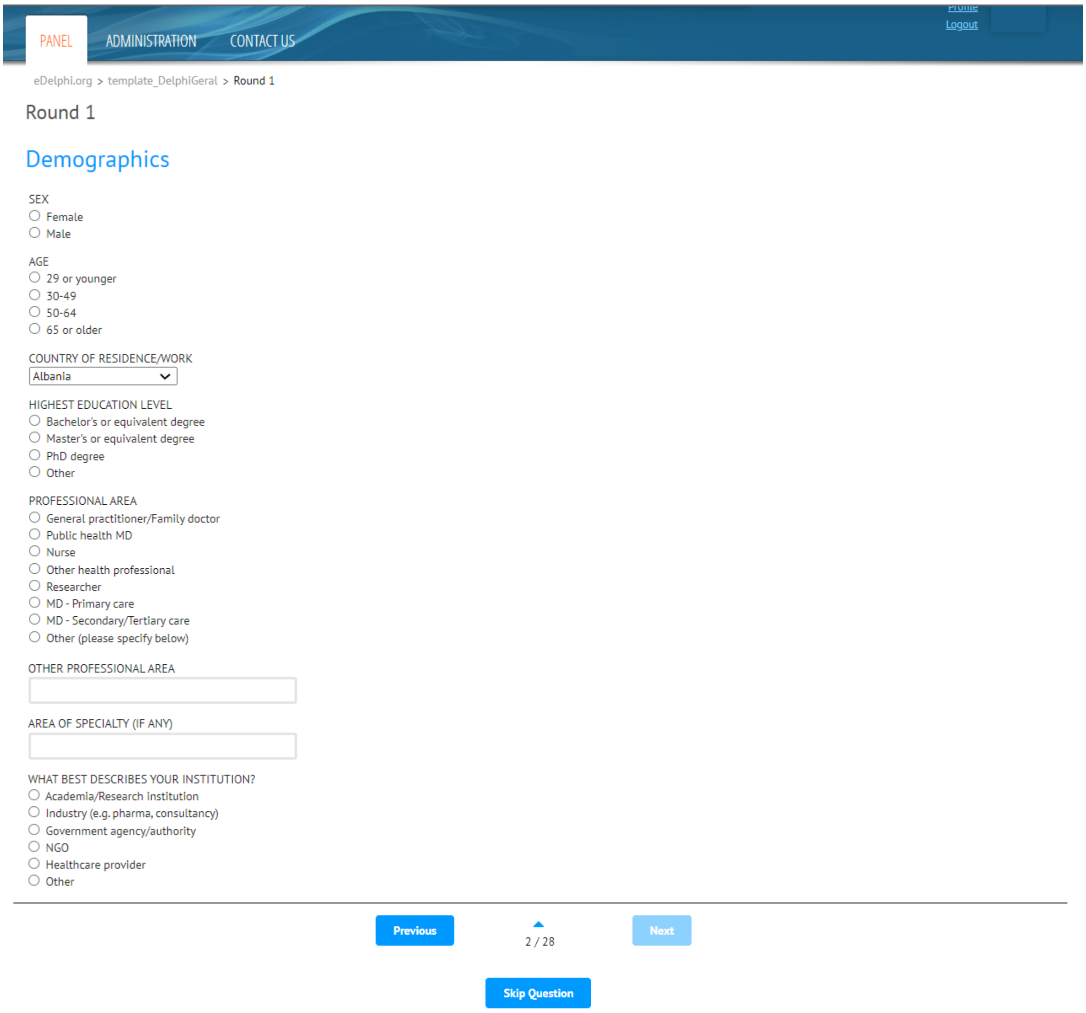
PANEL ADMINISTRATION CONTACT US

Profile
Logout

eDelphi.org > template_DelphiGeral > Round 1

Round 1

Demographics

SEX
- Female
- Male

AGE
- 29 or younger
- 30-49
- 50-64
- 65 or older

COUNTRY OF RESIDENCE/WORK
Albania

HIGHEST EDUCATION LEVEL
- Bachelor's or equivalent degree
- Master's or equivalent degree
- PhD degree
- Other

PROFESSIONAL AREA
- General practitioner/Family doctor
- Public health MD
- Nurse
- Other health professional
- Researcher
- MD - Primary care
- MD - Secondary/Tertiary care
- Other (please specify below)

OTHER PROFESSIONAL AREA

AREA OF SPECIALTY (IF ANY)

WHAT BEST DESCRIBES YOUR INSTITUTION?
- Academia/Research institution
- Industry (e.g. pharma, consultancy)
- Government agency/authority
- NGO
- Healthcare provider
- Other

Previous 2 / 28 Next

Skip Question

The remaining pages of the questionnaires present the QIs (one per page). Each QI, when applicable is characterized by a “Context”, “Name”, “Description”, “Formula”, “Calculation measure”, and “Sources”.

Round 1

Rate each indicator regarding the two criteria adopted in this Delphi study, using the radio buttons available for this purpose. At the bottom of the page, you may also leave comments/suggestions that you may find relevant to share with the other experts/participants.

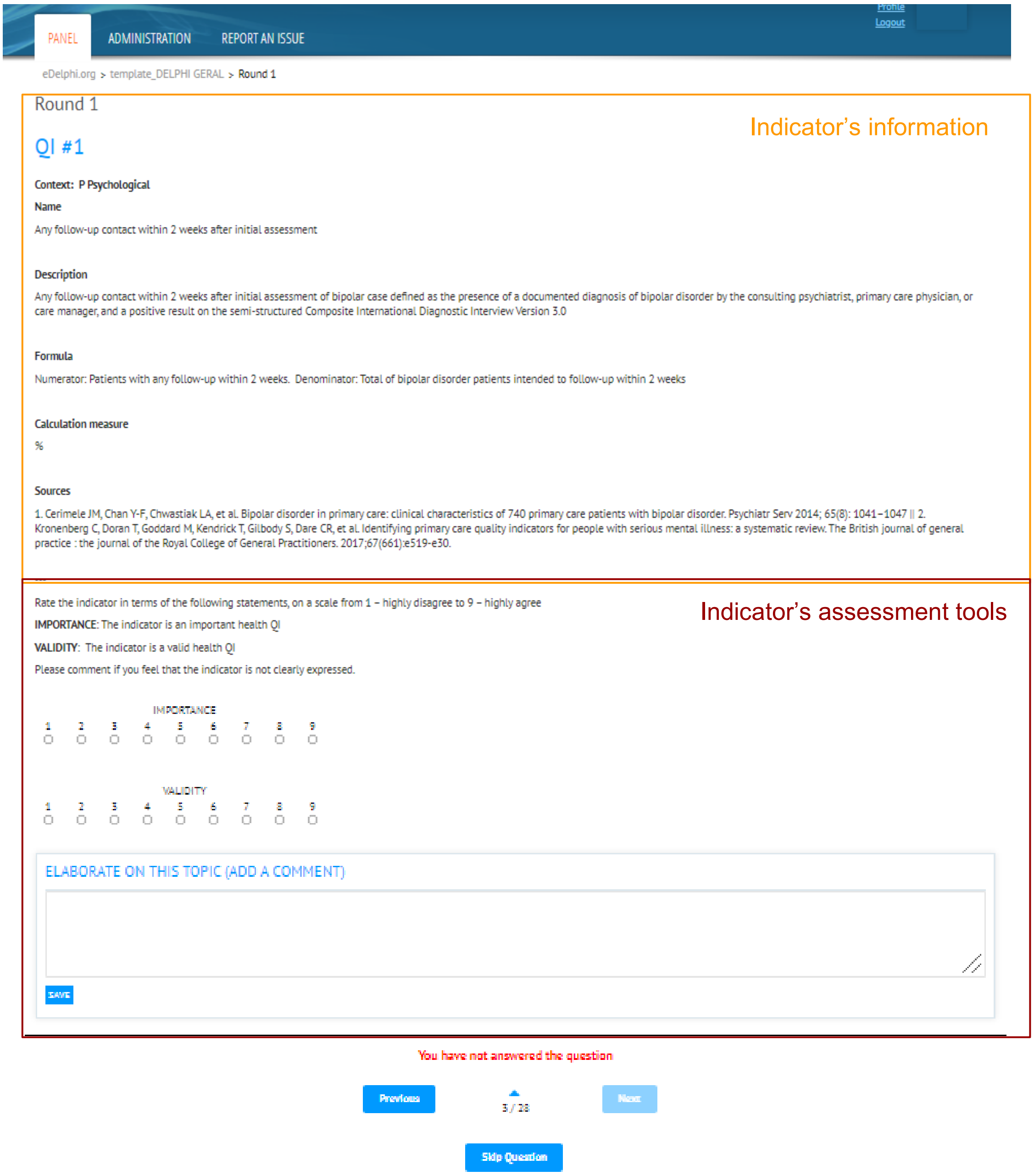

PANEL ADMINISTRATION REPORT AN ISSUE

Profile
Logout

eDelphi.org > template_DELPHI GERAL > Round 1

Round 1

Indicator's information

QI #1

Context: P Psychological

Name

Any follow-up contact within 2 weeks after initial assessment

Description

Any follow-up contact within 2 weeks after initial assessment of bipolar case defined as the presence of a documented diagnosis of bipolar disorder by the consulting psychiatrist, primary care physician, or care manager, and a positive result on the semi-structured Composite International Diagnostic Interview Version 3.0

Formula

Numerator: Patients with any follow-up within 2 weeks. Denominator: Total of bipolar disorder patients intended to follow-up within 2 weeks

Calculation measure

%

Sources

1. Cerimele JM, Chan Y-F, Chwastiak LA, et al. Bipolar disorder in primary care: clinical characteristics of 740 primary care patients with bipolar disorder. Psychiatr Serv 2014; 65(8): 1041–1047 || 2. Kronenberg C, Doran T, Goddard M, Kendrick T, Gilbody S, Dare CR, et al. Identifying primary care quality indicators for people with serious mental illness: a systematic review. The British journal of general practice : the journal of the Royal College of General Practitioners. 2017;67(661):e519-e30.

Indicator's assessment tools

Rate the indicator in terms of the following statements, on a scale from 1 – highly disagree to 9 – highly agree

IMPORTANCE: The indicator is an important health QI

VALIDITY: The indicator is a valid health QI

Please comment if you feel that the indicator is not clearly expressed.

IMPORTANCE

1 2 3 4 5 6 7 8 9

VALIDITY

1 2 3 4 5 6 7 8 9

ELABORATE ON THIS TOPIC (ADD A COMMENT)

SAVE

You have not answered the question

Previous 3 / 28 Next

Skip Question

Round 2

The questionnaire of Round 2 will include an additional information for each indicator. Besides the description of each indicator, it will be presented 1) the distribution of the group's ratings in the previous round, and, when available, 2) the comments provided before.

The goal of this information is to give you the group's perspective on each QI in the previous round.

QI #15

Indicator's Information

Context: W - Pregnancy and family planning

Name

Preventive care: Immunizable conditions

Description

Preventive care: Immunizable conditions (completed immunization)

Formula

Numerator: Number of patients in the denominator with completed immunization. Denominator: Total of prenatal patients followed

Calculation measure

Proportion

Sources

1. Kringos, D. S., Boerma, W. G., Hutchinson, A., van der Zee, J., & Groenewegen, P. P. (2010). The breadth of primary care: a systematic literature review of its core dimensions. BMC Health Services Research, 10(1) // 2. Marshall M, Klazinga N, Leatherman S, Hardy C, Bergmann E, Pisco L, et al: OECD Health Care Quality Indicator Project. The expert panel on primary care prevention and health promotion. Int J Qual Health Care 2006, 18(Suppl 1):21-25.

Ratings and comments from round 1

RESULTS FROM ROUND 1

1. Distribution of the group's ratings

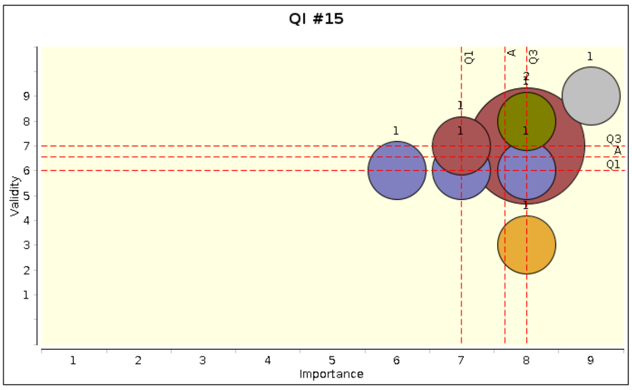

2. Comments

Comment #1: "It is unclear what the indicador is actually measuring, as preventive screening could encompass a wide range of conditions."

The ratings from round 1 will be summarized in a bubble plot, which provides reference lines for descriptive statistics (Q1, Median, Q3) and where the size of bubbles are proportional to the number of participants rating the same pair of values.

Moreover, Round 2 will be carried in real-time. This means that the ratings/comments of other experts that participated thus far in round 2 will be up-dated in real-time and made visible right after you answer. This method allows for participants to evaluate each indicator two times in the same round. The first assessment is made considering the results from the previous round only and a second assessment when the results of the current round become visible.

Rate each indicator regarding the two criteria adopted in this Delphi study by clicking with the mouse over the point that represents your combination of ratings on the coordinate grid provided for this purpose.

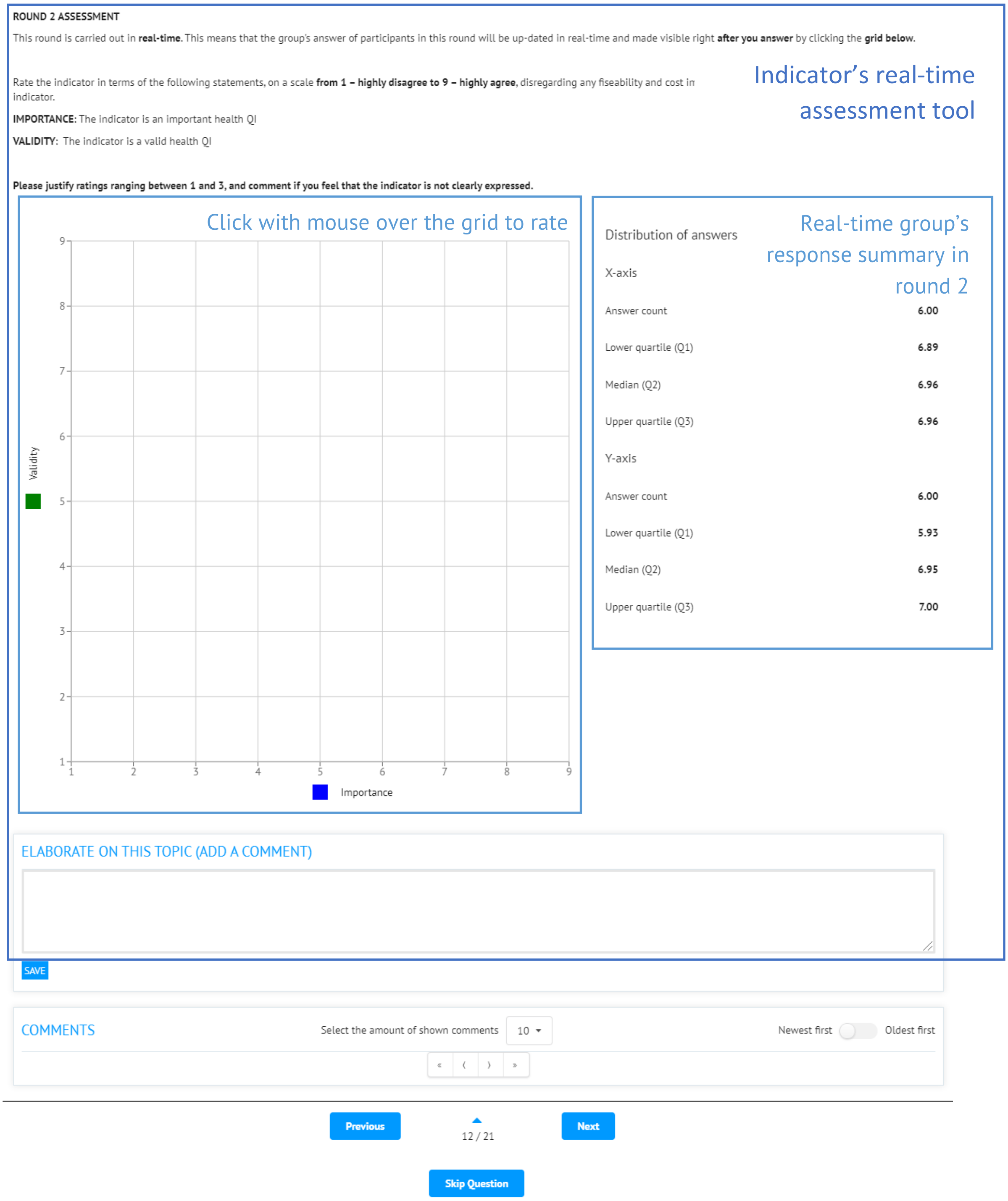

Supplement: S4 File — (PDF) [file pone.0309395.s005.pdf]
