# Supplementary material for: Selection of primary health care quality indicators in Europe: A Delphi study protocol
Source: PLoS One. 2024 Oct 24;19(10):e0309395. doi: 10.1371/journal.pone.0309395 (PMC11500873; doi:10.1371/journal.pone.0309395)
Supplement: S1 File — (DOCX) [file pone.0309395.s002.docx]

**Selection of primary health care quality indicators in Europe**: **a Delphi study** protocol

Supplementary material

**Table S2.1. Distribution of panels and their characteristics.**

| **Clinical context(s)** | **# of panels** | **# of indicators**  **(min-max)** |
| --- | --- | --- |
| W - Pregnancy and family planning / X - Female Genital / Y - Male Genital | 2 | 23-24 |
| D - Digestive tract | 1 | 33 |
| F - Eyes / H - Ears | 1 | 23 |
| R - Respiratory | 4 | 27-27 |
| T - Endocrine/Metabolic and Nutritional | 6 | 25-26 |
| K - Cardiovascular | 10 | 25-25 |
| U - Urological | 11 | 26-27 |
| L - Musculoskeletal | 3 | 23-23 |
| P - Psychological / N - Nervous system | 5 | 29-30 |
| A - General and unspecified / S - Skin | 11 | 27-28 |
| Not Defined | 3 | 24-24 |

Clinical contexts defined according to WHO ICPC-2 chapters categorization[ WONCA–World Organization of Family Doctors. An introduction to the international classification of primary care. Version 2" at http://www.ph3c.org/PH3C/docs/27/000098/0000054.pdf Accessed on 05 Jul 2019]

Table S2.2 List of European countries (**https://www.who.int/countries**).

| Albania | Greece | Portugal |
| --- | --- | --- |
| Andorra | Hungary | Republic of Moldova |
| Armenia | Iceland | Romania |
| Austria | Ireland | Russian Federation |
| Azerbaijan | Israel | San Marino |
| Belarus | Italy | Serbia |
| Belgium | Kazakhstan | Slovakia |
| Bosnia and Herzegovina | Kyrgyzstan | Slovenia |
| Bulgaria | Latvia | Spain |
| Croatia | Lithuania | Sweden |
| Cyprus | Luxemburg | Switzerland |
| Czech Republic | Malta | Tajikistan |
| Denmark | Monaco | Türkiye |
| Estonia | Montenegro | Turkmenistan |
| Finland | Netherlands | Ukraine |
| France | North Macedonia | United Kingdom |
| Georgia | Norway | Uzbekistan |
| Germany | Poland |  |

D.S2.1. Initial invitation to participate in the study

Subject: How to assess the quality of primary health care? Collaborate in a Delphi study

Dear Dr. <NAME>,

Health indicators are essential to assess, compare and improve the quality of health care services. The lack of a validated and common list of indicators hinders the benefits from these tools to guide strategies for the promotion of health and the prevention of diseases.

To address this challenge, we would like to invite **general practitioners and public health physicians, nurses, and other healthcare professionals, as well as researchers and other stakeholders in healthcare quality assessment**, to take part in a Delphi study to choose the main quality indicators to assess the quality of primary health care delivery **in European countries**.

Using a snowball approach to reach as many experts as possible, recruitment will strategically start through the members of the EUPHA - European Public Health Association and WONCA Europe - World Organization of Colleges, Academies and Academic Associations of **Family and Public Health Doctors and Nursing**, as well as through editors and the **associate editors** of journals publishing work in related fields of research.

This Delphi study will be conducted within the scope of a Portuguese R&D project entitled ***“***[***1st.IndiQare***](https://cintesis.eu/en/portfolio-items/1st_indiqare/) ***- Quality indicators in primary health care: validation and implementation of quality indicators as an assessment and comparison tool”***.

Please join our effort by participating in this Delphi study and sharing this invitation to contact emails of specialists in your country and other European countries.

Every participation will be greatly appreciated and will contribute to a better understanding of primary care quality needs across Europe. In this sense, we encourage you **to read the information about this study available in the participant consent form**, as well as to **please forward this message to all your mailing lists of colleagues** with the above-mentioned profile residing/working in European countries**.**

Best regards,

Mariana Lobo<https://orcid.org/0000-0003-3890-7735>

(on behalf of the 1st.IndiQare team)

**MEDCIDS** – Department of Community Medicine, Information and Health Decision Sciences

**CINTESIS** - Center for Health Technology and Services Research

D.S2.2. Invitation email to participate in Round 1 of a specific panel

Dear Expert,

Thank you for your interest and consent to collaborate in the 1st.IndiQare Delphi study to define the main and most consensual indicators to assess the quality of primary health care delivery.

The process will be done through an online program called eDelphi, which allows user-friendly, anonymous participation, as well as interrupting and resuming according to your own preferences.

The list of candidate quality indicators has been distributed across several Delphi panels. Each panel will comprise approximately 10 participants who will have to assess between 20 to 50 indicators. You will only take part in one of these panels. We anticipate on average three-minutes of your time per indicator to complete each round. This study has been approved by the CHSJ/FMUP Ethics Committee for Health of the Hospital Center of São João/Faculty of Medicine of the University of Porto.

Please find below the link to accept/decline your participation in the panel “[PANEL NAME]”.

In order to allow a timely conclusion of the study, we would respectfully request a response time of 2 weeks for the completion of each round. There are no right or wrong answers as the study is seeking for your opinion. We hope you will find the process interesting and the results will be made available to you at the end of the study.

To ACCEPT, please click the link below:

[ACCEPT URL]

To DECLINE, please click the link below:

[DECLINE URL]

You will be able to see the quality indicators that were assigned to you in this panel after accepting the invitation. Note that you can still use the decline link after accepting to participate if you realize that you cannot participate in the panel, you won't get any more e-mail notifications about the panel. If you decline an invitation by accident, you can still accept it by clicking the link for acceptance.

Please notice that the links are personal! Do NOT share them with others.

eDelphi privacy policy is fully GDPR (EU General Data Protection Regulation 2016/679) and US Privacy Law compliant (see https://www.edelphi.org/privacypolicy.page).

Best Regards,

[SENDER NAME]

(on behalf of the 1st.IndiQare team)

Figure S2.1. Detail of the presentation of results of round 1 (distribution of ratings and comments) in the questionnaire of round 2.
